# A cross-sectional survey of knowledge regarding sodium-glucose co-transporter 2 inhibitors (SGLT2is) among primary care physicians and pharmacists in China

**DOI:** 10.1186/s40780-025-00508-4

**Published:** 2025-11-17

**Authors:** Yu Dong, Shunfu Zheng, Wenjing Wang, Deping Wang, Hongliang Wang, Caiying Xiang

**Affiliations:** 1 People’s Hospital of Kaihua, Quzhou City, Zhejiang Province China; 2 Kaihua County Center for Disease Control and Prevention, Quzhou City, Zhejiang Province China

**Keywords:** Sodium-glucose cotransporter-2 inhibitors, SGLT2i, Primary care physicians, Clinical rational use

## Abstract

**Background:**

Sodium-glucose cotransporter-2 inhibitors (SGLT2is) occupy a pivotal role in the management of Type 2 diabetes due to their unique glucose-lowering mechanism and multiple clinical benefits. Physicians, as decision-makers in treatment, and pharmacists, as disseminators of drug knowledge, play crucial roles in ensuring the effectiveness and safety of treatments involving SGLT2is. However, the extent of primary healthcare workers’ understanding of SGLT2i characteristics prior to usage remains unclear.

**Aim:**

This study aims to evaluate the level of knowledge pertaining to the use of SGLT2is among primary care physicians and hospital pharmacists.

**Methods:**

A field survey on the cognition of SGLT2is was conducted among internal medicine, surgical, general practice, and pharmacy departments within healthcare institutions and community health service centers in Kaihua County, Zhejiang Province, China. An online questionnaire, including demographic information and knowledge about SGLT2is, was designed and administered.

**Results:**

The survey included 105 physicians and 31 pharmacists, with 59.56% being male and 66.18% holding intermediate or senior professional titles. Scores were predominantly in the range of 9–11 out of a maximum of 16 points, encompassing 102 participants (75.00%), while 9 individuals (11.76%) scored 13 points or higher, and 13 participants (9.56%) scored 8 points or lower. Notably, 33.09% correctly identified that SGLT2is are not applicable for Type 1 diabetes patients, 15.44% correctly noted the need to discontinue SGLT2is before major surgery, 23.53% correctly answered that SGLT2is are not approved in China for treating Type 2 diabetes in children and adolescents, and 47.06% correctly recognized that SGLT2is can be combined with metformin to enhance glucose-lowering effects. Additionally, the average score of physicians in surgical departments was significantly lower than other departments (H = 19.733, *P* < 0.001), and female participants had significantly higher average scores than males (Z = -3.528, *P* < 0.001).

**Conclusion:**

Primary care physicians and pharmacists in Kaihua County exhibit a robust understanding of the mechanisms of SGLT2is. However, there are notable deficiencies in the management of perioperative care in surgery, in discerning indications across internal and general medicine, in identifying contraindications in special populations, and in the pharmacists’ roles in prescription review. These findings suggest a need for targeted educational initiatives to enhance understanding and safe use of SGLT2 inhibitors at the primary-care level.

## Introduction

Type 2 diabetes mellitus (T2DM), recognized as a global public health issue, has seen an escalating prevalence that imposes substantial burdens on both patients and society [1, 2]. Effective glycemic control can not only enhance the quality of life for patients but also significantly reduce the risk of complications, thereby alleviating long-term healthcare costs [3]. Among various antidiabetic agents, sodium-glucose cotransporter-2 inhibitors (SGLT2is) have emerged as a novel therapeutic class in the treatment of T2DM due to their unique mechanism of action and clinical benefits [4–9].

SGLT2is function by inhibiting the reabsorption of glucose in the kidneys, thereby increasing urinary glucose excretion and reducing blood glucose levels. Beyond their glycemic control effects, SGLT2is also offer benefits such as weight reduction, blood pressure lowering, and cardiovascular protection [10–16]. Currently, the use of SGLT2i extends beyond endocrinology to include cardiology and nephrology, among other specialties, where they have demonstrated numerous advantages. Nevertheless, the appropriate use of these agents depends critically on physicians’ accurate understanding of indications, adverse reactions, and special considerations for specific patient groups [17, 18]. Although existing studies, such as those by Gao et al., have noted differences in SGLT2i knowledge among physicians across specialties, showing higher awareness in endocrinology and general practice compared to relative unfamiliarity in internal medicine, these studies have not encompassed physicians in surgical departments or hospital pharmacists [19]. Indeed, while surgeons do not typically prescribe SGLT2is, their knowledge of medication management during the perioperative period is crucial; similarly, hospital pharmacists play a key role in prescription review and medication education, with their level of expertise directly impacting the rational use of medications. Therefore, this study includes both surgical department physicians and hospital pharmacists in its survey, aiming to gain a more comprehensive understanding of the current state of knowledge regarding SGLT2is among different healthcare professionals in primary care settings, to inform future training and interventions [20–23].

## Methods

### Study design and sampling

In this cross-sectional study, a stratified random sampling technique was employed. Initial stratification was based on job positions, with a preset minimum of 25 participants per stratum. This figure was derived from a pilot study, which showed a standard deviation of approximately 1.5 points. Given a ε of 0.3 and an α of 0.05, the calculated minimum sample size was 98, which was increased by 20% to account for potential non-responses, resulting in at least 120 participants. Random selection was conducted using random number generation in Excel from a staff roster. Participants were drawn in equal proportions (1:1:1) from internal medicine (excluding endocrinology), surgical departments (including general surgery, neurosurgery, emergency surgery, and orthopedics), and pharmacy. General practitioners from nine community health service centers were stratified separately, with a minimum of 30 participants drawn from this group. The final sample comprised 34 internal medicine physicians, 31 surgical department physicians, 31 pharmacists, and 40 general practitioners, totaling 136 participants. The online survey was conducted from April 1 to May 31, 2023. All participants provided informed consent as part of the electronic survey questionnaire. This study was approved by the Ethics Committee of Kaihua County People’s Hospital.

### Design of the questionnaire

The questionnaire was developed based on an extensive literature review and consultations with experts [12, 15]. A preliminary survey was conducted prior to the main survey. Based on the results of the preliminary survey, the questionnaire was revised to enhance its practicality, comprehensibility, and completeness. It took an average of 6 min to complete the questionnaire, which comprised two sections: the first section collected basic information about the respondents, such as age, gender, professional title, years of work experience, and educational background. The second section assessed knowledge about SGLT2is, covering topics such as the mechanism of action, indications, dosage and administration, adverse reactions, considerations for special populations, and drug interactions, with a total of 16 questions. Each question had a definitive correct or incorrect answer, with correct answers scored as 1 point, and “uncertain” responses counted as incorrect and not scored.

### Statistical analysis

Data were cleaned and analyzed using R software, version 4.3.3. Descriptive statistics were utilized to summarize the characteristics of all variables. Given the non-normal distribution of the data (Shapiro-Wilk test, *P* < 0.001), non-parametric tests were used for comparisons between groups: the Mann-Whitney U test for comparing continuous variables between two groups, and the Kruskal-Wallis H test for comparisons among three or more groups. Categorical variables (e.g., accuracy rate of knowledge-based questions) were compared using the Chi-square test or Fisher’s exact test as appropriate. All tests were considered statistically significant at a p-value of less than 0.05. No correction for multiple comparisons was applied; therefore, subgroup analyses should be interpreted as exploratory.

## Results

### Demographic data

As shown in Table 1,A total of 136 questionnaires were distributed, with a response rate of 100%. As shown in Table 1, the respondents included pharmacists and physicians from various departments and with diverse professional titles. The specific distribution was as follows: 31 pharmacists; 105 physicians, among whom there were 34 internal medicine physicians, 31 surgical physicians, and 40 general practitioners. Of these, 55 (40.44%) were female and 81 (59.56%) were male; 126 respondents (92.79%) held a bachelor’s degree or higher; 70 (51.47%) had over 10 years of professional experience; 90 (66.18%) held intermediate or senior professional titles.

**Table 1:** Comparison of baseline characteristics among different survey participants

Variable	*n* (%)	Score	Statistic	*P*
Total	136 (100)	10.000 (9.000, 11.000)		
**Sex**			Z=-3.528	< 0.001
Male	81 (59.559)	10.000 (9.000, 11.000)		
Female	55 (40.441)	10.000 (10.000, 11.500)		
**Age**			H = 3.767	0.152
25–35	60 (44.118)	10.000 (10.000, 11.000)		
35–50	51 (37.500)	10.000 (9.000, 11.000)		
≥ 50	25 (18.382)	10.000 (9.000, 10.000)		
**Department**			H = 19.773	< 0.001
Internal Medicine	34 (25.000)	10.000 (9.250, 11.000)		
General Practice	40 (29.412)	10.000 (9.750, 11.000)		
Surgery	31 (22.794)	9.000 (9.000, 10.000)		
Pharmacy	31 (22.794)	11.000 (10.000, 12.000)		
**Professional title**			H = 3.228	0.199
Resident doctor	46 (33.824)	10.000 (10.000, 11.000)		
Attending physician	49 (36.029)	10.000 (9.000, 11.000)		
Senior physician	41 (30.147)	10.000 (9.000, 11.000)		
**Educational background**			H = 1.479	0.477
Associate degree	10 (7.353)	10.000 (10.000, 10.750)		
Undergraduate degree	123 (90.441)	10.000 (9.000, 11.000)		
Master’s degree and doctorate	3 (2.206)	11.000 (10.500, 11.000)		
**Length of service (years)**			H = 3.626	0.163
0–5	23 (16.912)	10.000 (10.000, 11.000)		
5–10	43 (31.618)	10.000 (9.000, 11.000)		
> 10	70 (51.471)	10.000 (9.000, 11.000)		
**Ever prescribed SGLT-2i**			H = 6.485	0.039
Physicians not prescribing	29 (21.324)	10.000 (9.000, 11.000)		
Physicians prescribing	76 (55.882)	10.000 (9.000, 11.000)		
Pharmacists	31 (22.794)	11.000 (10.000, 12.000)		

Z: Mann-Whitney test, H: Kruskal-Wallis test

### Questionnaire scores

**Fig. 1:**
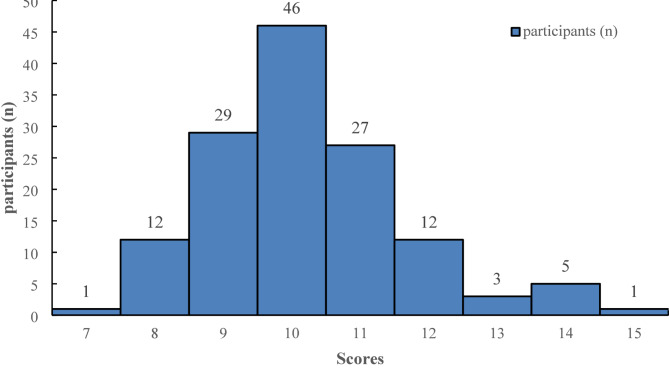
Distribution of knowledge scores regarding SGLT2i inhibitors among primary care physicians and pharmacists in rural healthcare settings

As shown in Fig. 1; Table 1, the maximum score possible on the questionnaire was 16. The study included 136 participants, with scores primarily ranging between 9 and 11 points, accounting for 102 individuals (75.00%). Nine participants (6.62%) scored 13 points or higher, while 13 participants (9.56%) scored 8 points or lower. Statistical analysis revealed that the median score in the surgical departments was significantly lower at 9 points compared to other departments (H = 19.733, *p* < 0.001). Female participants scored significantly higher than their male counterparts (Z = -3.528, *p* < 0.001); however, no significant differences were observed in scores when comparing age, professional title, educational background, or years of service. Excluding surgeons from the analysis did not significantly alter the median scores or subgroup patterns (shown in Table 2).

**Table 2:** Comparison of baseline characteristics among different survey participants (Excluding Surgeons)

Variable	*n* (%)	Score	Statistic	*P*
Total	105 (100)	10.000 (10.000, 11.000)		
**Sex**			Z=-1.823	0.068
Male	53 (50.476)	10.000 (9.000, 11.000)		
Female	52 (49.524)	10.000 (10.000, 12.000)		
**Age**			H = 3.421	0.181
25–35	46 (43.810)	10.000 (10.000, 11.000)		
35–50	42 (40.000)	10.000 (9.000, 11.000)		
≥ 50	17 (16.190)	10.000 (9.000, 11.000)		
**Department**			H = 2.322	0.313
Internal Medicine	34 (32.381)	10.000 (9.250, 11.000)		
General Practice	40 (38.095)	10.000 (9.750, 11.000)		
Surgery	31 (29.524)	11.000 (10.000, 12.000)		
Pharmacy			H = 0.517	0.772
**Professional title**	40 (38.095)	10.000 (10.000, 11.000)		
Resident doctor	37 (35.238)	10.000 (10.000, 11.000)		
Attending physician	28 (26.667)	10.000 (9.000, 11.000)		
Senior physician			H = 0.073	0.964
**Educational background**	10 (9.524)	10.000 (10.000, 10.750)		
Associate degree	93 (88.571)	10.000 (9.000, 11.000)		
Undergraduate degree	2 (1.905)	10.500 (10.250, 10.750)		
Master’s degree and doctorate			H = 2.139	0.343
**Length of service (years)**	19 (18.095)	10.000 (10.000, 11.500)		
0–5	32 (30.476)	10.000 (10.000, 11.250)		
5–10	54 (51.429)	10.000 (9.000, 11.000)		
> 10			H = 2.075	0.354
**Ever prescribed SGLT-2i**	17 (16.190)	10.000 (10.000, 11.000)		
Physicians not prescribing	57 (54.286)	10.000 (9.000, 11.000)		
Physicians prescribing	31 (29.524)	11.000 (10.000, 12.000)		
Pharmacists	105 (100)	10.000 (10.000, 11.000)		

Z: Mann-Whitney test, H: Kruskal-Wallis test

### Knowledge mastery

As shown in Table 3, the questions with a correct response rate of ≥ 80% and their specific scores are as follows. 96.32% (131/136) of participants correctly understood the cardiovascular protective effects of SGLT2is. 94.85% (129/136) accurately answered that SGLT2is reduce blood glucose by increasing urinary glucose excretion. 86.76% (118/136) correctly identified that SGLT2is aid in weight reduction. 89.71% (122/136) correctly responded to the effect of SGLT2is in reducing proteinuria. 82.35% (112/136) accurately answered that SGLT2is increase the risk of genitourinary tract infections. 88.97% (121/136) correctly responded that SGLT2is can induce ketoacidosis. 82.35% (112/136) accurately answered that adequate water intake should be encouraged during SGLT2i therapy. 94.12% (128/136) correctly answered that SGLT2i dosage needs adjustment in patients with severe liver dysfunction.

For questions with a correct response rate of < 60%, the specifics are as follows. 58.82% (80/136) of participants correctly identified the SGLT2i variants currently in use at our institution. Only 34.56% (47/136) correctly answered that SGLT2is have a protective effect on renal function in patients with mild to moderate renal dysfunction. Furthermore, 14.71% (20/136) of participants accurately responded that SGLT2is are not first-line medications for the initial treatment of type 2 diabetes. 33.09% (45/136) correctly recognized that SGLT2is are not applicable for the treatment of Type 1 diabetes mellitus. 15.44% (21/136) correctly pointed out that patients should discontinue SGLT2is before major surgery. 23.53% (32/136) correctly answered that SGLT2is are not approved in China for treating Type 2 diabetes in children and adolescents. 47.06% (64/136) correctly responded that SGLT2is can be used in combination with metformin to enhance the hypoglycemic effect.

Different medical specialties exhibited varying deficiencies in their knowledge of sodium-glucose SGLT2is. Among internal medicine physicians, 44.12% were aware of the specific SGLT2i brands used in the hospital; 21.59% correctly stated that SGLT2is are not considered first-line drugs for initial therapy; 26.47% correctly answered that SGLT2is are not approved in China for treating Type 2 diabetes in children and adolescents; and 5.88% correctly identified the necessity of discontinuing SGLT2is before major surgery. Among general practitioners, 27.5% correctly responded that SGLT2is are not suitable for treating Type 1 diabetes; 13.5% of the physicians correctly acknowledged that SGLT2is are not first-line medications for initial treatment; 12.5% were aware that SGLT2is are not approved for pediatric and adolescent Type 2 diabetes treatment in China; and 40% correctly stated that SGLT2is can be combined with metformin to enhance glucose-lowering effects. Among surgeons, 3.23% were aware of the specific SGLT2i brands available in the hospital; 16.13% understood the renal protective effect of SGLT2is in patients with mild to moderate renal impairment; and 9.68% correctly noted the importance of discontinuing SGLT2is before major surgery. Among pharmacists, 35.84% correctly recognized that SGLT2is are not suitable for treating Type 1 diabetes; 35.48% had accurate knowledge of medication management before major surgery; and 48.39% understood the contraindications for pediatric use.

**Table 3:** Comparison of multidimensional knowledge levels regarding SGLT2i among healthcare professionals in different clinical departments [N (%) of correct responses]

Questions	Internal medicine *N* = 34	General practices *N* = 40	Surgical department *N* = 31	Pharmacy department *N* = 31	Total *N* = 136	χ2	*P*
1. (Yes/no) Our hospital currently stocks a variety of SGLT2i agents, including dapagliflozin, empagliflozin, canagliflozin, and ertugliflozin.	15(44.12)	37(92.50)	1(3.23)	27(87.10)	80(58.82)	71.557	< 0.001
2. (Yes/no) SGLT2i has a cardiovascular protective effect and can reduce the risk of cardiovascular events.	33(97.06)	40(100.00)	30(96.77)	28(90.32)	131(96.32)	4.749	0.191
3. (Yes/no) SGLT2i has no protective effect on renal function in patients with mild to moderate renal insufficiency.	16(47.06)	11(27.50)	5(16.13)	15(48.39)	47(34.56)	5.188	0.159
4. (Yes/no) SGLT2i reduces blood glucose levels by increasing urinary glucose excretion.	31(91.18)	37(92.50)	30(96.77)	31(100.00)	129(94.85)	3.311	0.346
5. (Yes/no) SGLT2i is effective in reducing blood glucose and body weight.	28(82.35)	34(85.00)	30(96.77)	26(83.87)	118(86.76)	3.615	0.306
6. (Yes/No) SGLT2i can reduce proteinuria in patients with type 2 diabetes.	32(94.12)	36(90.00)	29(93.55)	25(80.65)	122(89.71)	3.972	0.265
7. (Yes/No) SGLT2i can be used to treat patients with type 1 diabetes.	16(47.06)	11(27.50)	7(22.58)	11(35.48)	45(33.09)	10.507	0.015
8. (Yes/No) SGLT2i is now used as a first-line drug for the initial management of type 2 diabetes.	7(21.59)	5(13.50)	3(9.69)	5(16.13)	20(14.71)	1.768	0.622
9. (Yes/no) Patients who use SGLT2i do not have an increased risk of genitourinary tract infections.	29(85.29)	33(82.50)	26(83.87)	24(77.42)	112(82.35)	0.771	0.856
10. (Yes/No) SGLT2i does not induce ketoacidosis.	29(85.29)	39(97.50)	27(87.10)	26(83.87)	121(88.97)	4.366	0.225
11. (Yes/No) SGLT2i use does not cause fatigue or low energy.	24(70.59)	32(80.00)	29(93.55)	22(70.97)	107(78.68)	6.553	0.088
12. (Yes/no) Adequate water intake should be encouraged in patients during SGLT2i therapy.	31(91.18)	29(72.50)	27(87.10)	25(80.65)	112(82.35)	5.036	0.169
13. (Yes/No) In patients with type 2 diabetes and severe hepatic dysfunction, SGLT2i dose adjustment is not necessary.	33(97.06)	38(95.00)	28(90.32)	29(93.55)	128(94.12)	4.749	0.191
14. (Yes/no) Before major surgery, patients should continue to take SGLT2i without discontinuing it.	2(5.88)	5(12.50)	3(9.68)	11(35.48)	21(15.44)	12.971	0.005
15. (Yes/no) Currently, SGLT2i has been approved for the treatment of type 2 diabetes in children and adolescents in China.	9(26.47)	5(12.50)	3(9.68)	15(48.39)	32(23.53)	16.819	< 0.001
16. (Yes/no) SGLT2i can be used in combination with metformin to enhance the hypoglycemic effect.	23(67.65)	16(40.00)	10(32.26)	15(48.39)	64(47.06)	9.332	0.025

## Discussion

This study conducted a cross-sectional survey that unveiled a dual characteristic in the understanding of SGLT2is among physicians and pharmacists in Kaihua County, characterized by a clear foundational knowledge but weak application in practice. Specifically, the vast majority of participants demonstrated an accurate grasp of the core pharmacological mechanisms of SGLT2is, with 94.85% correctly describing their glucosuric and weight-reducing effects, and 96.32% aware of their capacity to lower the risk of hospitalization for heart failure, indicating effective dissemination of basic benefits of these medications. However, there is a significant deficiency in the depth of knowledge required for clinical application: only 33.09% of respondents mastered the overall indications, 23.53% could identify contraindications in children and adolescents, and a mere 15.44% were aware of the protocols for discontinuing these drugs perioperatively. Notably, physicians in surgical departments displayed significantly lower levels of knowledge across all dimensions of clinical depth compared to their counterparts in other specialties. These findings suggest that primary healthcare professionals in Kaihua County have established a preliminary understanding of the basic pharmacology of SGLT2is. Nevertheless, there is a considerable gap in critical clinical decision-making capabilities, such as selecting appropriate indications, recognizing contraindications in special populations, and managing perioperative risks, which urgently necessitates enhancement through systematic training and the development of clinical pathways.

The findings of this study demonstrate that medical professionals in Kaihua County possess a relatively superior level of awareness regarding SGLT2is. Specifically, the scores of respondents from Kaihua County were concentrated between 9 and 11 out of a possible 16 points, equating to an accuracy rate of 62.5%. This performance surpasses the average level reported in studies of Jordanian community pharmacists, who scored an average of 6.61 out of 12 points (55.1% accuracy) [24], and also exceeds the average scores of general practitioners and trainee physicians in Belgium, who achieved 7.14 out of 15 points (47.6% accuracy) [25]. Although direct comparisons with Saudi studies are challenging due to differences in assessment tools and the number of questions, the scores obtained there (2.22 out of 4 points, 55.5% accuracy) [26] also reveal a notable discrepancy in knowledge levels compared to those in Kaihua County. A cross-sectional online survey conducted by Liu et al. involving 358 internists and general practitioners across 57 hospitals in Beijing indicated that only 39.1% (140 physicians) had a moderate understanding of the mechanisms and clinical applications of SGLT2is; those who claimed to have a comprehensive understanding comprised only 14.2% (51 physicians), while 17.9% (55 physicians) admitted to having almost no knowledge, and an additional 22.6% (81 physicians) reported having only superficial knowledge [27]. These figures are relatively consistent with our findings. However, unlike studies focused solely on community pharmacists in Jordan, general practitioners and trainee physicians in Belgium, and internists and general practitioners in Beijing, this research encompasses both surgeons and pharmacists in its evaluative framework. It reveals a critical deficiency in the understanding of perioperative safety management of SGLT2is among surgeons, identifying specific, high-risk areas of insufficient knowledge concealed within the overall superior performance. These insights provide empirical evidence for enhancing perioperative safety for diabetic patients in Kaihua County.

Our study revealed significant gaps in primary care physicians’ understanding of the indications for SGLT2is, combination drug therapies, and guidelines for pediatric medication. Previous research has highlighted that a lack of knowledge about indications can lead to inappropriate prescribing practices, while insufficient understanding of drug interactions may increase the risk of adverse reactions in patients [28, 29]. Furthermore, the U.S. Food and Drug Administration (FDA) has approved drugs such as empagliflozin for improving glycemic control in children aged 10 years and older with Type 2 diabetes [30]. However, in China, the drug labels do not authorize the use of SGLT2is for children and adolescents, which could potentially increase legal risks associated with treatment. Additionally, some physicians may not be fully aware of the contraindications of SGLT2is in specific populations, such as patients with severe renal impairment or those at risk of urinary tract infections [29]. Moreover, inadequate awareness of potential adverse effects, such as genitourinary infections and diabetic ketoacidosis (DKA), could lead to these conditions being overlooked or misdiagnosed [31, 32].

As China progressively implements a tiered system for chronic disease management, primary healthcare services are playing an increasingly vital role in comprehensive diabetes prevention and control [33]. Internal medicine and general practitioners in primary healthcare settings are key players in this process, and their professional competence directly impacts the quality of diabetes management [34, 35].

Although this study demonstrates that medical personnel in Kaihua County possess a sound understanding of the basic mechanisms of SGLT2is, the current findings also reveal significant gaps in their knowledge regarding the clinical applications of these drugs. Such deficiencies may challenge the precise administration of these pharmacological agents.

For example, only 36.49% of the surveyed physicians demonstrated a proper understanding of the indications for SGLT2is, and knowledge about the contraindications for using SGLT2is in children was even lower, at just 18.92%.

Furthermore, although physicians in surgical departments do not routinely prescribe SGLT2is, their awareness of the protocols for discontinuing these medications during the perioperative period is crucial to ensure that the drugs are completely eliminated from the patient’s system before surgery. Failure to discontinue SGLT2is in a timely manner perioperatively could induce DKA, a serious side effect [36]. Symptoms of DKA induced by SGLT2is may be atypical, and blood glucose levels may not be significantly elevated, making it easy to overlook. Research indicates that about 50% of DKA cases during SGLT2i therapy are associated with certain triggers, such as dehydration, physical stress, or planned surgery [37]. A survey indicates that only 9.68% of surgeons correctly recognize the need to discontinue SGLT2is preoperatively, suggesting that most are insufficiently aware of this risk. Additionally, emergency physicians encountering patients treated with SGLT2is and presenting with symptoms of metabolic acidosis need to be vigilant, promptly recognize the condition, and provide appropriate treatment [38]. Regardless of whether a patient’s blood glucose levels are elevated, it is imperative to immediately test for ketone bodies and conduct blood gas analysis to determine the potential development of DKA [37]. This scenario reveals a fundamental lack of interdisciplinary collaboration mechanisms: the management of SGLT2is is viewed as the responsibility of endocrinology specialists and has not been effectively integrated into surgical pathway management and emergency response protocols, leading to “departmental barriers” in the application of knowledge.

The role of pharmacists in prescription review cannot be overlooked, particularly in the management of complex diseases such as diabetes and heart disease. They play a crucial role by educating patients and physicians, optimizing medication therapy plans, reducing the risk of medical errors, and enhancing patient adherence to treatment [39, 40]. However, our survey revealed that pharmacists’ knowledge of medications is not ideal, with a significant portion of pharmacists incorrectly answering questions related to drug indications, contraindications for use in adolescents and children, and discontinuation perioperatively. Previous studies have shown that systematic drug training and public education significantly improve the medication knowledge of both physicians and patients [41]. Through pharmacological education and management interventions, pharmacists not only can improve patients’ medication adherence but also significantly enhance both clinical and non-clinical outcomes [42–44].

In conclusion, we propose the following recommendations: Firstly, targeted training programs should be initiated at the departmental level, with a specific emphasis on enhancing the education of surgical physicians in perioperative pharmacological management. Existing research underscores the significant impact that training programs and educational initiatives can potentially have on improving the pharmacological expertise of both physicians and pharmacists [45].

Secondly, Clinical Decision Support Systems (CDSS) serve as essential adjunct tools for physicians prescribing medications and pharmacists conducting prescription reviews. Integrating CDSS into hospital computerized physician order entry (CPOE) systems—through predefined rules and algorithms—provides real-time, personalized reminders and advice at the point of prescribing. There is substantial evidence indicating that well-designed CDSS can significantly improve healthcare providers’ adherence to guidelines, optimize prescribing practices, and ultimately enhance patient outcomes [46, 47]. Notably, a systematic review focusing on the prescription of antidiabetic medications has shown that CDSS can effectively improve prescribing behaviors, increase guideline compliance, and moderately enhance intermediate outcomes such as glycemic control [48]. Lastly, the establishment of collaborative mechanisms between pharmacists and physicians, led by pharmacists conducting prescription reviews and patient education, can optimize medication therapy management. These measures will contribute to enhancing the proficiency of primary care professionals in key knowledge areas of widely used medications such as SGLT2is, ultimately improving patient treatment outcomes.

This study’s strength lies in its use of an independent questionnaire survey, ensuring that physicians and pharmacists reflect their knowledge of SGLT2is without external information, thus effectively revealing their understanding of the medication in real clinical practice. However, the design of true/false questions in the questionnaire may have allowed for some degree of random guessing, which could affect the accuracy of the results. Additionally, the limited sample size might introduce statistical bias. Future research should aim to expand the sample size and include a more diverse array of question types to enhance the generalizability of the findings. Furthermore, conducting cognitive surveys on medications widely used across disciplines and associated with potential severe adverse reactions to evaluate healthcare professionals’ understanding of critical drug knowledge could improve clinical medication safety and optimize patient treatment outcomes.

## Conclusion

Physicians and pharmacists in Kaihua County exhibit a commendable understanding of the mechanisms of SGLT2is. However, gaps exist in the perioperative management by surgical physicians, as well as in the mastery of indications and recognition of contraindications in special populations by internal medicine and general practitioners, alongside the clinical application knowledge of pharmacists. These deficiencies highlight the urgent need to improve medical professionals’ grasp of critical knowledge concerning widely used clinical medications. This finding suggests the necessity for precise training and informatics interventions targeted at core departmental scenarios to comprehensively enhance the level of safe and rational medication use at the primary care level.

## Data Availability

The datasets generated and analyzed during the current study are not publicly available in order to protect participant privacy. For questions regarding data, please contact Caiying Xiang (27453751@qq.com).

## Abbreviations

SGLT2i: Sodium glucose cotransporter-2 inhibitors
T2DM: Type 2 diabetes mellitus
FDA: Food and Drug Administration
DKA: Diabetic ketoacidosis

## References

[1] Sun H, Saeedi P, Karuranga S, Pinkepank M, Ogurtsova K, Duncan BB, et al. IDF diabetes atlas: Global, regional and country-level diabetes prevalence estimates for 2021 and projections for 2045. Diabetes Res Clin Pract. 2022;183:109119. 34879977 10.1016/j.diabres.2021.109119 PMC11057359

[2] Collaborators GBDD. Global, regional, and National burden of diabetes from 1990 to 2021, with projections of prevalence to 2050: a systematic analysis for the global burden of disease study 2021. Lancet. 2023;402:203–34. 37356446 10.1016/S0140-6736(23)01301-6 PMC10364581

[3] Parker ED, Lin J, Mahoney T, Ume N, Yang G, Gabbay RA, et al. Economic costs of diabetes In the U.S. In 2022. Diabetes Care. 2024;47:26–43. 37909353 10.2337/dci23-0085

[4] Zinman B, Wanner C, Lachin JM, Fitchett D, Bluhmki E, Hantel S, et al. Empagliflozin, cardiovascular Outcomes, and mortality in type 2 diabetes. N Engl J Med. 2015;373:2117–28. 26378978 10.1056/NEJMoa1504720

[5] Perkovic V, Jardine MJ, Neal B, Bompoint S, Heerspink HJL, Charytan DM, et al. Canagliflozin and renal outcomes in type 2 diabetes and nephropathy. N Engl J Med. 2019;380:2295–306. 30990260 10.1056/NEJMoa1811744

[6] Neal B, Perkovic V, Mahaffey KW, de Zeeuw D, Fulcher G, Erondu N, et al. Canagliflozin and cardiovascular and renal events in type 2 diabetes. N Engl J Med. 2017;377:644–57. 28605608 10.1056/NEJMoa1611925

[7] Wiviott SD, Raz I, Bonaca MP, Mosenzon O, Kato ET, Cahn A, et al. Dapagliflozin and cardiovascular outcomes in type 2 diabetes. N Engl J Med. 2019;380:347–57. 30415602 10.1056/NEJMoa1812389

[8] Heerspink HJL, Stefansson BV, Correa-Rotter R, Chertow GM, Greene T, Hou FF, et al. Dapagliflozin in patients with chronic kidney disease. N Engl J Med. 2020;383:1436–46. 32970396 10.1056/NEJMoa2024816

[9] Packer M, Anker SD, Butler J, Filippatos G, Pocock SJ, Carson P, et al. Cardiovascular and renal outcomes with empagliflozin in heart failure. N Engl J Med. 2020;383:1413–24. 32865377 10.1056/NEJMoa2022190

[10] Vaduganathan M, Docherty KF, Claggett BL, Jhund PS, de Boer RA, Hernandez AF, et al. SGLT-2 inhibitors in patients with heart failure: a comprehensive meta-analysis of five randomised controlled trials. Lancet. 2022;400:757–67. 36041474 10.1016/S0140-6736(22)01429-5

[11] Nuffield Department of Population Health Renal Studies G, Consortium SiM-AC-RT. Impact of diabetes on the effects of sodium glucose co-transporter-2 inhibitors on kidney outcomes: collaborative meta-analysis of large placebo-controlled trials. Lancet. 2022;400:1788–801. 36351458 10.1016/S0140-6736(22)02074-8 PMC7613836

[12] Li S, Vandvik PO, Lytvyn L, Guyatt GH, Palmer SC, Rodriguez-Gutierrez R, et al. SGLT-2 inhibitors or GLP-1 receptor agonists for adults with type 2 diabetes: a clinical practice guideline. BMJ. 2021;373:n1091. 33975892 10.1136/bmj.n1091

[13] Cowie MR, Fisher M. SGLT2 inhibitors: mechanisms of cardiovascular benefit beyond glycaemic control. Nat Rev Cardiol. 2020;17:761–72. 32665641 10.1038/s41569-020-0406-8

[14] Zelniker TA, Braunwald E. Mechanisms of cardiorenal effects of sodium-glucose cotransporter 2 inhibitors: JACC state-of-the-art review. J Am Coll Cardiol. 2020;75:422–34. 32000955 10.1016/j.jacc.2019.11.031

[15] O’Hara DV, Lam CSP, McMurray JJV, Yi TW, Hocking S, Dawson J, et al. Applications of SGLT2 inhibitors beyond glycaemic control. Nat Rev Nephrol. 2024;20:513–29. 38671190 10.1038/s41581-024-00836-y

[16] Palmer SC, Tendal B, Mustafa RA, Vandvik PO, Li S, Hao Q, et al. Sodium-glucose cotransporter protein-2 (SGLT-2) inhibitors and glucagon-like peptide-1 (GLP-1) receptor agonists for type 2 diabetes: systematic review and network meta-analysis of randomised controlled trials. BMJ. 2021;372:m4573. 33441402 10.1136/bmj.m4573 PMC7804890

[17] Pasquel FJ, Lansang MC, Dhatariya K, Umpierrez GE. Management of diabetes and hyperglycaemia in the hospital. Lancet Diabetes Endocrinol. 2021;9:174–88. 33515493 10.1016/S2213-8587(20)30381-8 PMC10423081

[18] Li S, Yu C, Li Y, Li Q, Zhang R, Hou Q, et al. Study design and baseline characteristics of inpatients with diabetes mellitus in a tertiary hospital in China: a database study based on electronic medical records. J Evid Based Med. 2018;11:152–7. 29512333 10.1111/jebm.12291

[19] Gao Y, Peterson E, Pagidipati N. Barriers to prescribing glucose-lowering therapies with cardiometabolic benefits. Am Heart J. 2020;224:47–53. 32304879 10.1016/j.ahj.2020.03.017

[20] Libianto R, Davis TM, Ekinci EI. Advances in type 2 diabetes therapy: a focus on cardiovascular and renal outcomes. Med J Aust. 2020;212:133–9. 31910303 10.5694/mja2.50472

[21] Peters AL, McGuire DK, Danne T, Kushner JA, Rodbard HW, Dhatariya K, et al. Diabetic ketoacidosis and related events with sotagliflozin added to insulin in adults with type 1 diabetes: a pooled analysis of the intandem 1 and 2 studies. Diabetes Care. 2020;43:2713–20. 32928957 10.2337/dc20-0924 PMC7576419

[22] Palanca A, van Nes F, Pardo F, Ampudia Blasco FJ, Mathieu C. Real-world evidence of efficacy and safety of SGLT2 inhibitors as adjunctive therapy in adults with type 1 diabetes: a European two-center experience. Diabetes Care. 2022;45:650–8. 35061022 10.2337/dc21-1584

[23] Osafehinti DA, Okoli OJ, Karam JG. A case of SGLT2 inhibitor-associated euglycemic diabetic ketoacidosis following coronary artery bypass surgery. AACE Clin Case Rep. 2021;7:20–2. 33851014 10.1016/j.aace.2020.11.014 PMC7924150

[24] Alqudah A, Oqal M, Al-Samdi A, Qnais E, Wedyan M, Abu Gneam M, et al. Knowledge and practice of community pharmacists towards SGLT2 inhibitors. F1000Res. 2022;11:659. 35811806 10.12688/f1000research.122170.1 PMC9237554

[25] Lecomte J, de Beeck IO, Mamouris P, Mathieu C, Goderis G. Knowledge and prescribing behaviour of flemish general practitioners regarding novel glucose-lowering medications: online cross-sectional survey. Prim Care Diabetes. 2024;18:441–7. 38862313 10.1016/j.pcd.2024.06.002

[26] Somaili M, Oraibia O, Darraj M, Hassan A, Moafa E, Kulaybi A, et al. Assessment of knowledge and perception of sodium-glucose co-transporter 2 (SGLT-2) inhibitors prescription among physicians in Saudi Arabia. Curr Diabetes Rev. 2024;20:e060723218471. 37415371 10.2174/1573399820666230706125244

[27] Liu J, Su X, Hao Y, Liu J. Cardio metabolic survey i. Knowledge gap and prescribing patterns of glucagon-like peptide-1 receptor agonists and sodium-glucose cotransporter 2 inhibitors among Chinese Doctors. Sci Rep. 2024;14:18290. 39112571 10.1038/s41598-024-69016-z PMC11306237

[28] Zwart K, Velthuis S, Polyukhovych YV, Mosterd A, Smidt L, Serne EH, et al. Sodium-glucose cotransporter 2 inhibitors: a practical guide for the dutch cardiologist based on real-world experience. Neth Heart J. 2021;29:490–9. 34132981 10.1007/s12471-021-01580-9 PMC8455761

[29] Ng NM, Ng YS, Chu TK, Lau P. Factors affecting prescription of sodium-glucose co-transporter 2 inhibitors in patients with type 2 diabetes mellitus with established cardiovascular disease/ chronic kidney disease in Hong Kong: a qualitative study. BMC Prim Care. 2022;23:317. 36476327 10.1186/s12875-022-01928-z PMC9730654

[30] Furth SL. Trials and tribulations - the challenges of clinical trials in children. NEJM Evid. 2023;2:EVIDe2300280. 38320507 10.1056/EVIDe2300280

[31] Bersoff-Matcha SJ, Chamberlain C, Cao C, Kortepeter C, Chong WH. Fournier gangrene associated with sodium-glucose cotransporter-2 inhibitors: a review of spontaneous postmarketing cases. Ann Intern Med. 2019;170:764–9. 31060053 10.7326/M19-0085

[32] Alliabi FJA, Jaber AAS, Jallo MKI, Baig MR. Adherence of physicians to evidence-based management guidelines for treating type 2 diabetes and atherosclerotic cardiovascular disease in Ajman, United Arab Emirates. BMC Prim Care. 2022;23:70. 35392814 10.1186/s12875-022-01672-4 PMC8988318

[33] Lu J, Yang H, Shi L, Sheng X, Huo Y, Liu R, et al. Associations between primary healthcare experiences and glycemic control status in patients with diabetes: results from the greater bay area study, China. Int J Environ Res Public Health. 2023;20. 10.3390/ijerph20021120 PMC9859184 36673874

[34] Yao J, Wang H, Yin J, Shao D, Guo X, Sun Q, et al. Factors associated with the utilization of community-based diabetes management care: a cross-sectional study in Shandong Province, China. BMC Health Serv Res. 2020;20:407. 32393254 10.1186/s12913-020-05292-5 PMC7212576

[35] Tao X, Mao L, Zhang P, Ma X, Liang Z, Sun K, et al. Barriers and facilitators to primary care management of type 2 diabetes in Shijiazhuang City, china: a mixed methods study. BMC Prim Care. 2024;25:84. 38481166 10.1186/s12875-024-02330-7 PMC10935988

[36] Seki H, Ideno S, Shiga T, Watanabe H, Ono M, Motoyasu A, et al. Sodium-glucose cotransporter 2 inhibitor-associated perioperative ketoacidosis: a systematic review of case reports. J Anesth. 2023;37:465–73. 36849747 10.1007/s00540-023-03174-8 PMC10229478

[37] Musso G, Saba F, Cassader M, Gambino R. Diabetic ketoacidosis with SGLT2 inhibitors. BMJ. 2020;371:m4147. 33184044 10.1136/bmj.m4147

[38] Yamamoto M, Ide N, Kitajima S, Obayashi M, Asada K, Matsushima S, et al. Risk of euglycemic diabetic ketoacidosis due to low-carbohydrate diet while taking empagliflozin: a case report. Yakugaku Zasshi. 2019;139:1479–83. 31685745 10.1248/yakushi.19-00120

[39] Tanaka A, Node K. Emerging roles of sodium-glucose cotransporter 2 inhibitors in cardiology. J Cardiol. 2017;69:501–7. 28043708 10.1016/j.jjcc.2016.10.019

[40] Traynor K. Pharmacy projects target SGLT2 inhibitors for heart failure. Am J Health Syst Pharm. 2023;80:404–5. 36815338 10.1093/ajhp/zxad042

[41] Shi W, Qin H, Vaughan B, Ng L. Educational interventions for medical students to improve pharmacological knowledge and prescribing skills: a scoping review. Perspect Med Educ. 2023;12:348–60. 37662713 10.5334/pme.1006 PMC10473179

[42] Wilhelmsen NC, Eriksson T. Medication adherence interventions and outcomes: an overview of systematic reviews. Eur J Hosp Pharm. 2019;26:187–92. 31338165 10.1136/ejhpharm-2018-001725 PMC6613929

[43] Syrnyk M, Glass B. Pharmacist interventions in medication adherence in patients with mental health disorders: a scoping review. Int J Pharm Pract. 2023;31:449–58. 37319335 10.1093/ijpp/riad037

[44] Ahmed A, Guo P, Jalal Z. A systematic review investigating the role and impact of pharmacist interventions in cardiac rehabilitation. Int J Clin Pharm. 2023;45:320–9. 36401764 10.1007/s11096-022-01517-1 PMC10147760

[45] Wenning L, Pillai GC, Knepper TC, Ilic K, Ali AM, Hibma JE. Clinical pharmacology worldwide: a global health perspective. Clin Pharmacol Ther. 2021;110:946–51. 33893656 10.1002/cpt.2274

[46] Sutton RT, Pincock D, Baumgart DC, Sadowski DC, Fedorak RN, Kroeker KI. An overview of clinical decision support systems: benefits, risks, and strategies for success. NPJ Digit Med. 2020;3:17. 32047862 10.1038/s41746-020-0221-y PMC7005290

[47] Page N, Baysari MT, Westbrook JI. A systematic review of the effectiveness of interruptive medication prescribing alerts in hospital CPOE systems to change prescriber behavior and improve patient safety. Int J Med Inf. 2017;105:22–30. 10.1016/j.ijmedinf.2017.05.011 28750908

[48] Tlili NE, Robert L, Gerard E, Lemaitre M, Vambergue A, Beuscart JB, et al. A systematic review of the value of clinical decision support systems in the prescription of antidiabetic drugs. Int J Med Inf. 2024;191:105581. 10.1016/j.ijmedinf.2024.105581 39106772

